# The Association Between Visceral Obesity and Postoperative Outcomes in Elderly Patients With Colorectal Cancer

**DOI:** 10.3389/fsurg.2022.827481

**Published:** 2022-06-06

**Authors:** Qiantong Dong, Haonan Song, Weizhe Chen, Wenbin Wang, Xiaojiao Ruan, Tingting Xie, Dongdong Huang, Xiaolei Chen, Chungen Xing

**Affiliations:** ^1^ Department of General Surgery, The Second Affiliated Hospital of Soochow University, Suzhou, China; ^2^ Department of Gastrointestinal Surgery, The First Affiliated Hospital of Wenzhou Medical University, Wenzhou, China; ^3^ Department of Gastrointestinal Surgery, Shanghai Tenth People’s Hospital Affiliated to Tongji University, Tongji University School of Medicine, Shanghai, China

**Keywords:** colorectal cancer, elderly patients, postoperative complication, visceral obesity, risk factor

## Abstract

**Background:**

The impact of visceral obesity on the postoperative complications of colorectal cancer in elderly patients has not been well studied. This study aims to explore the influence of visceral obesity on surgical outcomes in elderly patients who have accepted a radical surgery for colorectal cancer.

**Methods:**

Patients aged over 65 year who had undergone colorectal cancer resections from January 2015 to September 2020 were enrolled. Visceral obesity is typically evaluated based on visceral fat area (VFA) which is measured by computed tomography (CT) imaging. Univariate and multivariate analyses were performed to analyze parameters related to short-term outcomes.

**Results:**

A total of 528 patients participated in this prospective study. Patients with visceral obesity exhibited the higher incidence of total (34.1% vs. 18.0%, *P *< 0.001), surgical (26.1% vs. 14.6%, *P* = 0.001) and medical (12.6% vs. 6.7%, *P* = 0.022) complications. Based on multivariate analysis, visceral obesity and preoperative poorly controlled hypoalbuminemia were considered as independent risk factors for postoperative complications in elderly patients after colorectal cancer surgery.

**Conclusions:**

Visceral obesity, evaluated by VFA, was a crucial clinical predictor of short-term outcomes after colorectal cancer surgery in elderly patients. More attentions should be paid to these elderly patients before surgery.

## Introduction

The incidence and mortality of colorectal cancer (CRC) have been significantly increasing in China during the past decade, ranking the third most common cancer (1). Globally, with the population becoming older in recent years, a larger number of elderly patients were diagnosed with CRC, comprising the majority of the population with CRC (2). Till now, radical resection remains the only curative treatment for CRC. Also, an increasing number of colorectal resections are being performed on more and more elderly patients. Elderly patients with CRC tend to have multiple comorbidities, decreased social and cognitive functioning, poor nutritional status, and worse prognosis such as worse overall and disease-free survival (3). In addition, they are more prone to developing morbidity and mortality after surgery as advancing age reduces physiological reserve capacity to deal with major abdominal surgery (4–6). There is still some controversy about the surgical treatment for elderly patients with CRC. Some studies indicated that elderly patients with CRC had higher postoperative complication rates than younger population (7, 8). Therefore, the surgeons must weigh the benefits of radical colorectal resection for elderly patients. Moreover, highly precise geriatric assessments to identify patients at higher risks for developing adverse outcomes are required (9–11).

Aging is also associated with dramatic changes in body fat distribution. A main concern in the aging society is the increasing prevalence of obesity, which is known as a risk factor for cancer and physical dysfunction (12). Visceral obesity is characterized by excessive amounts of intra-abdominal adipose tissue accumulation (13). Visceral obesity has been shown previously to increase the risk of surgical procedures, which may increase postoperative complication rates and hospital stay (14–16).

Colorectal cancer is well known as an “obesity-related” cancer (17, 18). Epidemiological studies indicated that there was a substantial association between the incidence of CRCs and visceral obesity (19). In recent years, emerging evidence suggests that visceral obesity is closely related to poor prognosis after colorectal surgery (15, 20, 21). The impact of visceral obesity has been demonstrated in the general population, but there is limited knowledge for the elderly population.

In this study, we investigated whether visceral obesity would predict short-term outcomes in elderly patients after resection for colorectal cancer.

## Methods and Materials

### Patients

This retrospective study was approved by the Ethics Committee of The First Affiliated Hospital of Wenzhou Medical University. Between January 2015 and September 2020, patients aged 65 and over who underwent elective resection for colorectal cancer in Gastrointestinal Surgical Department, the First Affiliated Hospital of Wenzhou Medical University were included. Inclusion criteria were patients who (1) had an accurate diagnosis of colorectal cancer on the basis of histological evidence before surgery; (2) were medically fit for surgery (American Society of Anesthesiologists [ASA] grade ≤III); (3) had preoperative computed tomography (CT) scans to measure abdominal VFA (within 1 month before surgery). Exclusion criteria were: (1) patients who were accepted for palliative resection or emergency surgery; (2) patients who received neoadjuvant chemotherapy or radiotherapy. All surgical procedures were performed by 4 surgeons who were highly experienced in radical colorectal resections for colorectal cancer (more than 150 cases).

### Data Collection

Patient information, including patient characteristics, operative details, and postoperative short-term outcomes, were obtained from our clinical information system. Patient characteristics, collected within 1 month before surgery, comprised age, gender, body mass index (BMI), visceral fat area (VFA), plasma albumin concentration (hypoalbuminemia was defined as a plasma albumin concentration of less than 35 g/L), hemoglobin concentration (anemia is defined as a hemoglobin concentration of less than 120 g/L in men or 110 g/L in women), American Society of Anesthesiology (ASA) grade, preoperative nutritional risk screening 2002 (NRS 2002) scores (22), comorbidity (assessed by Charlson comorbidity Index score) (23), history of previous abdominal surgery, tumor location, and tumor-node-metastasis (TNM) stage. The parameters of operative details were laparoscopic surgery, number of dissected lymph nodes (at least 15 lymph nodes were dissected), positive lymph node, surgical durations, and combined resection. Postoperative short-term outcomes consisted of postoperative complications (during hospital stays or within 30 days after operation), postoperative hospital stays, hospitalization cost, and readmissions within 30 days of discharge. Postoperative complications were classified as grade II or higher based on the Clavien-Dindo classification system (24).

### Visceral Fat Area Measurement

Computed tomography (CT) axial slices taken at the third lumbar vertebra (L3) was used for visceral fat area (VFA) measurements. The boundaries of the adipose tissue were outlined on the CT image using standard Hounsfield unit ranges (−150 to −50 Hu), and then VFA was calculated. To minimize measurement bias, all measurements were completed by one radiologist who was blinded for the clinical details of the subjects on a dedicated processing system (version 3.0.11.3 BN17 32 bit; INFINITT Healthcare Co., Ltd). According to a previous study, visceral obesity was defined as a VFA > 130 cm^2^ in men and >90 cm^2^ in women (25). According to this parameter, patients were stratified into visceral obesity (VO) and Non-visceral obesity (Non-VO) groups.

### Statistical Analysis

Normally distributed data were described as mean value and standard deviation (SD). Nonnormally distributed data were presented as median value and interquartile range (IQR). Student’s t-test, Mann–Whitney U-test (or Kruskal–Wallis H test) and Chi-square test (or Fisher’s exact test) were used to compare normally distributed variables, nonnormally distributed variables and categorical variables respectively. The multivariate logistic regression or Cox proportional hazards regression analysis (forward stepwise selection processes) was used to determine the independent risk factor. *P* values <0.05 (two-tailed) was considered statistically significant, and all statistical analyses were performed using SPSS statistics version 22.0 (IBM, Armonk, New York, USA).

## Results

### Patients’ Characteristics

The demographic and clinicopathologic characteristics of 528 patients (261 in VO group vs. 267 in Non-VO group) included are summarized in Table 1. Mean age was 74.1 years old and 318 (60.2%) patients had male sex. The median VFA was 110.0 cm^2^ and 261 (49.4%) patients were defined as having visceral obesity (including 138 males and 123 females). 74 patients were diagnosed with diabetes. Additionally, women were more prone to have visceral obesity than men (*P* = 0.001). For the clinicopathological parameters, patients with visceral obesity had higher BMI (*P* < 0.001), higher albumin (*P* = 0.039), higher Charlson comorbidity index (*P* < 0.001), and lower NRS 2002 scores (*P* = 0.033). There were no significant differences in age, hemoglobin, previous abdominal surgery, and tumor location between the two groups. Regarding the operative parameters, the median numbers of dissected lymph nodes (25 in VO group vs. 31 in Non-VO group, *P* < 0.001) and the median surgical durations (180 min in VO group vs. 160 min n Non-VO group, *P* = 0.002) were significantly different. Moreover, two groups did not show significant differences regarding laparoscopy-assisted operation, positive lymph node and combined resection.

**Table 1:** Comparison of clinical characteristics between the VO group and the Non-VO group.

Variables	Total (*n* = 528)	VO group (*n* = 261)	Non-VO group (*n* = 267)	*P* value
Age, mean (SD), years	74.1 (6.2)	74.0 ± 6.0	74.1 ± 6.4	0.913
Gender				0.001*
Male	318 (60.2)	138 (52.9)	180 (67.4)	
Female	210 (39.8)	123 (47.1)	87 (32.6)	
BMI, mean (SD), kg/m^2^	22.5 ± 3.1	24.2 ± 2.7	20.9 ± 2.5	<0.001*
VFA, median (IQR), cm^2^	110.0 (90.0)	157.2 (73.7)	67.0 (54.7)	0.001*
Albumin, mean (SD), g/L	36.3 ± 4.4	36.7 ± 4.5	35.9 ± 4.3	0.039*
Hemoglobin, median (IQR), g/L	118.5 (20)	119 (20)	116 (20)	0.705
ASA grade				0.076
I	132 (25.0)	48 (18.4)	84 (31.5)	
II	321 (60.8)	182 (69.7)	139 (52.1)	
III	75 (14.2)	31 (11.9)	44 (16.5)	
NRS 2002 scores				0.033*
≥3	235 (44.5)	104 (39.8)	131 (49.1)	
<3	293 (55.5)	157 (60.2)	136 (50.9)	
Charlson comorbidity index^a^				<0.001*
0	222 (42.0)	81 (31.0)	141 (52.8)	
1	198 (37.5)	108 (41.4)	90 (33.7)	
≥2	108 (20.5)	72 (27.6)	36 (13.5)	
Previous abdominal surgery				0.972
Yes	117 (22.2)	58 (22.2)	59 (22.2)	
No	411 (77.8)	203 (77.8)	208 (77.9)	
Tumor location				0.214
Colon	285 (54.0)	148 (56.7)	137 (51.3)	
Rectum	243 (46.0)	113 (43.3)	130 (48.7)	
TNM stage				0.041*
I	96 (18.2)	54 (20.7)	42 (15.7)	
II	219 (41.5)	114 (43.7)	105 (39.3)	
III	195 (36.9)	82 (31.4)	113 (42.3)	
IV	18 (3.4)	11 (4.2)	7 (2.6)	
Laparoscopy-assisted operation				0.170
Yes	235 (44.5)	124 (47.5)	111 (41.6)	
No	293 (55.5)	137 (52.5)	156 (58.4)	
Numbers of dissected lymph nodes, median (IQR)	26 (5)	25 (6)	31 (5)	<0.001*
Lymph node positive	194 (36.7)	86 (33.0)	108 (40.4)	0.074
Combined resection				0.156
Yes	38 (7.2)	23 (8.8)	15 (5.6)	
No	490 (92.8)	238 (91.2)	252 (94.4)	

*Values in parentheses are percentages unless indicated otherwise.*

*SD, standard deviation; BMI, body mass index; VFA, visceral fat area; IQR, interquartile range; ASA, American Society of Anesthesiologists; NRS, nutritional risk screening; TNM, tumor–node–metastasis.*

^a^ *Charlson Comorbidity Index doesn’t consider points coming from cancer.*

* *P *<* 0.05 was considered statistically significant.*

### Postoperative Outcomes

As shown in Table 2, the overall incidence of postoperative complications was 25.9%. The most common complications were wound infection (*n* = 29, 5.5%), intra-abdominal abscess (*n* = 27, 5.1%) and anastomotic leakage (*n* = 25, 4.7%). The incidence of total complications was significantly higher in the VO group than that in the Non-VO group (34.1% vs. 18.0%, *P* < 0.001). Further analysis of the complications showed that in the VO group, both surgical (26.1% vs. 14.6%, *P *= 0.001), and medical (12.6% vs. 6.7%, *P *= 0.022) complications were more common than for patients in the non-VO group. Regarding the details of complications, VO patients experienced more wound infection (*P *= 0.030), intra-abdominal abscess (*P* = 0.025), and pulmonary complications (*P* = 0.023) than Non-VO patients. No case of mortality occurred. The median postoperative hospital stay was 13 days and 25 (4.7%) patients were readmitted within 30 days of discharge. No statistically significant differences were observed in postoperative hospital stay (*P* = 0.583), costs (*P* = 0.313) or readmission rate (*P* = 0.792) between the two groups.

**Table 2:** Short-term outcomes.

Factors	Total (*n* = 528)	VO group (*n* = 261)	Non-VO group (*n* = 267)	*P* value
Total complications	137 (25.9)	89 (34.1)	48 (18.0)	<0.001*
Surgical complications	107 (20.3)	68 (26.1)	39 (14.6)	0.001*
Gastrointestinal dysfunction^a^	5 (0.9)	3 (1.1)	2 (0.7)	0.980
Wound infection	29 (5.5)	20 (7.7)	9 (3.4)	0.030*
Bleeding	10 (1.9)	4 (1.5)	6 (2.2)	0.777
Intra-abdominal abscess	27 (5.1)	19 (7.3)	8 (3.0)	0.025*
Anastomotic leakage	25 (4.7)	16 (6.1)	9 (3.4)	0.136
Intestinal obstruction	11 (2.1)	6 (2.3)	5 (1.9)	0.732
Medical complications	51 (9.7)	33 (12.6)	18 (6.7)	0.022*
Pulmonary complications	17 (3.2)	13 (5.0)	4 (1.5)	0.023*
Cardiac complications	3 (0.6)	2 (0.8)	1 (0.4)	0.984
Venous thrombosis	9 (1.7)	5 (1.9)	4 (1.5)	0.973
Persistent hypoalbuminemia	20 (3.8)	12 (4.6)	8 (3.0)	0.335
Urinary infection	2 (0.4)	1 (0.4)	1 (0.4)	1.000
Reoperation for complications	4 (0.8)	1 (0.4)	3 (1.1)	0.632
Stoma	27 (5.1)	9 (3.4)	18 (6.7)	0.086
Surgical durations, median (IQR), minutes	170 (84)	180 (80)	160 (85)	0.002*
Postoperative hospital stays, median (IQR), days	13 (7)	13 (7.5)	13 (6)	0.583
Costs, median (IQR), yuan	54,892.3 (21,400.7)	55,593.0 (23,085.8)	53,753.7 (21,977.4)	0.313
Readmissions within 30 days of discharge	25 (4.7)	13 (5.0)	12 (4.5)	0.792

*Values in parentheses are percentages unless indicated otherwise.*

^a^ *Including prolonged postoperative ileus and diarrhea.*

* *P *<* 0.05 was considered statistically significant.*

### Univariate and Multivariate Analysis Associated with Complications

Potential risk factors for overall complications are listed in Table 3. In univariate analysis, higher BMI (*P* = 0.002), hypoalbuminemia (*P* = 0.034) and visceral obesity (*P* < 0.001) were associated with overall postoperative complications. The multivariate analysis revealed that hypoalbuminemia (OR: 1.692 (1.119–2.559), *P* = 0.013) and visceral obesity (OR: 2.482 (1.649–3.737), *P* < 0.001) remained as independent risk factors for overall complications. In terms of surgical complications, visceral obesity (OR: 2.060 (1.329–3.191), *P* = 0.001) was the unique independent risk factor (Table 4). As for medical complications, hypoalbuminemia (OR: 2.206 (1.224–3.977), *P* = 0.008) and visceral obesity (OR: 2.150 (1.170–3.953), *P* = 0.014) were independent risk factors (Table 5).

**Table 3:** Univariate and multivariate logistic regression analysis of risk factors for total complications.

Factors	Univariate analysis		Multivariate analysis	
	Case with complication (%)	*P*	OR (95% CI)	*P*
Age		0.079		
≥80/<80	38 (32.2)/99 (24.1)			
Gender		0.360		
Male/female	78 (24.5)/59 (28.1)			
BMI		0.002*		
<18.5	8 (16.3)			
18.5–24	73 (23.1)			
> 24	56 (34.4)			
Hypoalbuminemia		0.034*	1.692 (1.119–2.559)	0.013*
Yes/no	56 (31.6)/81 (23.1)			
Anemia		0.565		
Yes/no	55 (24.7)/82 (26.9)			
NRS 2002 scores		0.320		
≥3/<3	56 (23.8)/81 (27.6)			
ASA grade		0.314		
III/II, I	23 (30.7)/114 (25.2)			
Charlson comorbidity index		0.107		
0	51 (23.0)			
1	52 (26.3)			
≥2	34 (31.5)			
Visceral obesity		<0.001*	2.482 (1.649–3.737)	<0.001*
Yes/no	89 (34.1)/48 (18.0)			
Previous abdominal surgery		0.422		
Yes/no	27 (23.1)/110 (26.8)			
Tumor location		0.850		
Colon/rectum	73 (25.6)/64 (26.3)			
TNM stage		0.686		
I	25 (26.0)			
II	54 (24.7)			
III, IV	58 (27.2)			
Combined resection		0.411		
Yes/no	12 (31.6)/125 (25.5)			
Laparoscopic-assisted surgery		0.111		
Yes/no	53 (22.6)/84 (28.7)			

*OR, Odds Ratio; CI, Confidence Interval; BMI, Body mass index; ASA, American Society of Anesthesiologists; TNM, tumor–node–metastasis.*

* *Statistically significant (P < 0.05).*

**Table 4:** Univariate and multivariate logistic regression analysis of risk factors for surgical complications.

Factors	Univariate analysis		Multivariate analysis	
	Case with complication (%)	*P*	OR (95% CI)	*P*
Age		0.114		
≥80/<80	30 (25.4)/77 (18.8)			
Gender		0.100		
Male/female	57 (17.9)/50 (23.8)			
BMI		0.080		
<18.5	6 (12.2)			
18.5–24	62 (19.6)			
> 24	39 (23.9)			
Hypoalbuminemia		0.239		
Yes/no	41 (23.2)/66 (18.8)			
Anemia		0.358		
Yes/no	41 (18.4)/66 (21.6)			
NRS 2002 scores		0.568		
≥3/<3	45 (19.1)/62 (21.2)			
ASA grade		0.804		
III/II, I	16 (21.3)/91 (20.1)			
Charlson comorbidity index		0.379		
0	41 (18.5)			
1	42 (21.2)			
≥2	24 (22.2)			
Visceral obesity		0.001*	2.060 (1.329–3.191)	0.001*
Yes/no	68 (26.1)/39 (14.6)			
Previous abdominal surgery		0.333		
Yes/no	20 (17.1)/87 (21.2)			
Tumor location		0.703		
Colon/rectum	56 (19.6)/51 (21.0)			
TNM stage		0.720		
I	19 (19.8)			
II	43 (19.6)			
III, IV	45 (21.1)			
Combined resection		0.167		
Yes/no	11 (28.9)/96 (19.6)			
Laparoscopic-assisted surgery		0.568		
Yes/no	45 (19.1)/62 (21.2)			

*OR, Odds Ratio; CI, Confidence Interval; BMI, Body mass index; ASA, American Society of Anesthesiologists; TNM, tumor–node–metastasis.*

* *Statistically significant (P < 0.05).*

**Table 5:** Univariate and multivariate logistic regression analysis of risk factors for medical complications.

Factors	Univariate analysis		Multivariate analysis	
	Case with complication (%)	*P*	OR (95% CI)	*P*
Age		0.104		
≥80/<80	16 (13.6)/35 (8.5)			
Gender		0.932		
Male/female	31 (9.7)/20 (9.5)			
BMI		0.022		
<18.5	3 (6.1)			
18.5–24	25 (7.9)			
>24	23 (14.1)			
Hypoalbuminemia		0.014*	2.206 (1.224–3.977)	0.008*
Yes/no	25 (14.1)/26 (7.4)			
Anemia		0.463		
Yes/no	24 (10.8)/27 (8.9)			
NRS 2002 scores		0.615		
≥3/<	21 (8.9)/30 (10.2)			
ASA grade		0.113		
III/II, I	11 (14.7)/40 (8.8)			
Charlson comorbidity index		0.156		
0	19 (8.6)			
1	16 (8.1)			
≥2	16 (14.8)			
Visceral obesity		0.022*	2.150 (1.170–3.953)	0.014*
Yes/no	33 (12.6)/18 (6.7)			
Previous abdominal surgery		0.414		
Yes/no	9 (7.7)/42 (10.2)			
Tumor location		0.876		
Colon/rectum	27 (9.5)/24 (9.9)			
TNM stage		0.327		
I	8 (8.3)			
II	19 (8.7)			
III, IV	24 (11.3)			
Combined resection		0.636		
Yes/no	5 (13.2)/46 (9.4)			
Laparoscopic-assisted surgery		0.047*		
Yes/no	16 (6.8)/35 (11.9)			

*OR, Odds Ratio; CI, Confidence Interval; BMI, Body mass index; ASA, American Society of Anesthesiologists; TNM, tumor–node–metastasis.*

* *Statistically significant (P < 0.05).*

## Discussion

The present study revealed that visceral obesity was an independent risk factor for overall, surgical and medical postoperative complications. Also, there was a significant positive association between visceral obesity and the operation of surgery, as the elderly patients with visceral obesity had longer surgical durations and less numbers of lymph nodes harvested. Multiple studies have examined the relationship between visceral obesity and postoperative outcomes. However, there was no study focus on the special population such as elderly population, which was the strength of our study. In addition, we also compared the prognostic value between BMI and visceral obesity. We found that visceral obesity was a better predictor than BMI for postoperative complications in elderly patients after colorectal cancer surgery.

Recently, studies investigating the association between obesity and cancer had demonstrated that it was visceral obesity rather than generalized body fat significantly contributed to poor prognosis in patients with pancreatic cancer (26). A systematic review and meta-analysis demonstrated that visceral obesity resulted in higher morbidity, longer surgical time, and lower lymph node retrieval after colorectal cancer surgery (27), which was consistent with our findings. Furthermore, visceral obesity was shown to predict a negative prognosis after other forms of surgery, such as gastrectomy, pancreaticoduodenectomy, and nephrectomy (28–30). These findings indicated that visceral obesity could be a meaningful predictor of postoperative complications. Although previous studies suggested visceral obesity was associated with poor prognosis, the cut-off value for visceral fat area (VFA) has not been clearly defined. Since there is no standardized cut-off value for VFA, some western studies used top sex-specific quartile to define visceral obesity patients (31). Other western studies considered defining visceral obesity with VFA >163.8 cm^2^ in males and >80.1 cm^2^ in females as cut points coming from a white population undergoing gastrointestinal resection (32). As the body composition differs from distinct regions, the results of these study may not be applicable to Asian population. In Asians, the most commonly used sex-specific VFA cut-off points are 130 cm² for males and 90 cm² for females (25), which were very different from those used in western studies. Possible reasons for this difference may be different anthropometric and clinical characteristics between Asian and western population. In the present study, we used the latter cut-off value to define visceral obesity.

Excessive visceral fat may result in an increase in pro-inflammatory adipocytokines, such as TNF-α, and IL-6, and in the releases free fatty acids into blood, which would break the balance of the immune reaction (33). During the postoperative period, the poor immune system could lead to an increase in the risk of postoperative complications (34). What’s more, in this study, 74 patients were diagnosed with diabetes, counting for 14% of entire population. Visceral obesity could damage the insulin signaling pathway and be associated with insulin resistance, which would cause infectious complications, especially wound infection (35). Expanded visceral fat, correlated with visceral obesity, elevates the difficulty of surgery, which may increase operative time and blood loss, resulting in higher surgical complications rates (36). Furthermore, elderly patients tend to have multiple comorbidities, which may lead to a worse prognosis. For these reasons mentioned above, elderly patients with visceral obesity easily had poor prognosis.

The results of this study indicated that we cannot ignore the preoperative diagnosis and intervention of visceral obesity in elderly patients with CRC. For surgeons, when making an operation choice, elderly patients with visceral obesity should be more carefully considered. Previous studies concluded that physical exercise is beneficial for preventing abdominal fat accumulation (37, 38). What’s more, a retrospective cohort study reported that increased VFA could cause reduced lung function (39). This finding is in accordance with results presented in this study. In Table 2, it can be noticed that patients in VO group suffered more pulmonary complications than non-VO group. For elderly patients with visceral obesity, exercise therapy prior to colorectal surgery should be widely implemented to improve physical condition and pulmonary function.

In term of lymph nodes harvested, the result of the present study concluded that visceral obesity patients had less number of dissected lymph nodes, while there is no significant differences in the number of positive lymph node. A recent study revealed that visceral obesity patients were less likely to have metastatic lymph nodes involvement in colorectal cancer and gastric cancer (40–42). There may be two explanations for this finding. One hypothesis is that it is more difficult to harvest an appropriate number of lymph nodes in visceral obesity patients because the excessive amounts of intra-abdominal adipose tissue accumulation increased the difficulty of surgery and limited accessibility to some deep lymph nodes. Another possible explanation is distinct microenvironments. Previous studies demonstrated that visceral obesity may create a harsh microenvironment for CRC cells which suppresses the invasion and growth of cancer cells (43). Thus, further studies are needed to validate the relationship between visceral obesity and metastatic lymph node in elderly patients with CRC.

In the present study, hypoalbuminemia, suggesting a poor nutritional status, also proved to be an independent risk factor for overall and medical complications. Due to the low protein intake in the potential malignant process of elderly patients, hypoalbuminemia was frequently observed in elderly patients. A possible mechanism is that hypoalbuminemia is a marker of systemic immunoinflammatory response to surgery, malnutrition, and cancer cachexia. It can be a prognostic tool of postoperative complications (44). Therefore, early identification and intervention in elderly patient with CRC and hypoalbuminemia would reduce the rate of postoperative complications. In addition, hypoproteinemia can be treated with oral nutritional supplements or an intravenous infusion of albumin, which can be a part of pre-rehabilitation program before surgery.

There were several limitations in this study. First, as there were no consensus reference cut-offs of VFA for severe visceral obesity, it was not classified in this study. Second, there was no relevant data for prediabetes or dyslipidaemia. Third, the long-term prognosis was not analyzed in this study. Long-term follow-up data should be collected and analyzed in future studies as the research continues.

## Conclusions

In conclusion, the present study demonstrated that elderly patients with visceral obesity, evaluated by VFA, exhibited a higher rate of postoperative complications after resection for CRC. Moreover, preoperative poorly controlled hypoalbuminemia and visceral obesity were independent risk factors for overall and medical complications. Visceral obesity was an independent risk factor for surgical complications. Therefore, as the population gets older and more susceptible to obesity, visceral obesity can be a clinical predictor for CRC resection in elderly patients.

## Data Availability

The raw data supporting the conclusions of this article will be made available by the authors, without undue reservation.
